# Correlations and Timeliness of COVID-19 Surveillance Data Sources and Indicators ― United States, October 1, 2020–March 22, 2023

**DOI:** 10.15585/mmwr.mm7219e2

**Published:** 2023-05-12

**Authors:** Heather M. Scobie, Mark Panaggio, Alison M. Binder, Molly E. Gallagher, William M. Duck, Philip Graff, Benjamin J. Silk

**Affiliations:** ^1^Coronavirus and Other Respiratory Viruses Division, National Center for Immunization and Respiratory Diseases, CDC; ^2^Applied Physics Laboratory, Johns Hopkins University, Laurel, Maryland; ^3^Division of Healthcare Quality Promotion, National Center for Emerging and Zoonotic Infectious Diseases, CDC; ^4^Office of Public Health Data, Surveillance, and Technology, CDC.

When the U.S. COVID-19 public health emergency declaration expires on May 11, 2023, national reporting of certain categories of COVID-19 public health surveillance data will be transitioned to other data sources or will be discontinued; COVID-19 hospitalization data will be the only data source available at the county level (*1*). In anticipation of the transition, national COVID-19 surveillance data sources and indicators were evaluated for purposes of ongoing monitoring. The timeliness and correlations among surveillance indicators were analyzed to assess the usefulness of COVID-19–associated hospital admission rates as a primary indicator for monitoring COVID-19 trends, as well as the suitability of other replacement data sources. During April 2022–March 2023, COVID-19 hospital admission rates from the National Healthcare Safety Network (NHSN)^†^ lagged 1 day behind case rates and 4 days behind percentages of positive test results and COVID-19 emergency department (ED) visits from the National Syndromic Surveillance Program (NSSP). In the same analysis, National Vital Statistics System (NVSS) trends in the percentage of deaths that were COVID-19–associated, which is tracked by date of death rather than by report date, were observable 13 days earlier than those from aggregate death count data, which will be discontinued (*1*). During October 2020–March 2023, strong correlations were observed between NVSS and aggregate death data (0.78) and between the percentage of positive SARS-CoV-2 test results from the National Respiratory and Enteric Viruses Surveillance System (NREVSS) and COVID-19 electronic laboratory reporting (CELR) (0.79), which will also be discontinued (*1*). Weekly COVID-19 Community Levels (CCLs) will be replaced with levels of COVID-19 hospital admission rates (low, medium, or high) which demonstrated >99% concordance by county during February 2022–March 2023. COVID-19–associated hospital admission levels are a suitable primary metric for monitoring COVID-19 trends, the percentage of COVID-19 deaths is a timely disease severity indicator, and the percentages of positive SARS-CoV-2 test results from NREVSS and ED visits serve as early indicators for COVID-19 monitoring. Collectively, these surveillance data sources and indicators can support monitoring of the impact of COVID-19 and related prevention and control strategies as ongoing public health priorities.

Authorizations to collect certain categories of public health data will expire on May 11, 2023 (*1*), including national data on the percentage of positive SARS-CoV-2 test results from CELR (*2*); national reporting of aggregate case and death counts, which CDC compiles from official public health jurisdiction sources, will also be discontinued (*3*). CDC will transition to using provisional mortality data from NVSS as the primary data source on COVID-19 deaths (*4*) and to using SARS-CoV-2 test positivity data from NREVSS, an established sentinel network of more than 450 clinical, public health, and commercial laboratories (*5*). Finally, county COVID-19 hospital admission levels based on admission rates per 100,000 population will replace CCLs^§^ as a primary metric for COVID-19 monitoring. CCLs were first designed to assist communities and members of the public in making prevention decisions based on local context and unique needs (*6*).

Statistical measures were used to compare trends in moving 7-day averages for COVID-19 surveillance indicators during October 1, 2020–March 22, 2023, including cross-correlation, autocorrelation, pairwise correlations, and a geographic consistency metric at the state level. Daily averages were available for most data sources; weekly data were available for NVSS,^¶^ NREVSS, and aggregate case and death counts after a shift to weekly cadence in October 2022, with some jurisdictions continuing to report daily totals. Cross-correlation was used to estimate the lag (offset in days) in indicators relative to COVID-19–associated hospital admission rates** during April 1, 2022–March 22, 2023, by calculating Spearman’s correlation coefficients by state with reporting lags from −35 days to 35 days over a moving 12-week window; the lag that produced the highest mean correlation was selected. Lags were adjusted to obtain temporal alignment of indicators in subsequent analyses. Pairwise Spearman’s correlations were used to evaluate associations between indicators, and mean correlations were calculated and ranked. Spearman’s autocorrelations were used to assess the signal-to-noise ratio for each indicator (compared with itself, offset by 7 days). Geographic consistency was evaluated using a metric calculated by computing daily z-scores for each indicator, averaging these scores by U.S. state, and computing the standard deviation over all states. Surveillance indicators with lower values for the geographic consistency metric were less likely to have jurisdictions consistently reporting higher or lower than average indicator values.

A linear regression model^††^ was fit for each surveillance indicator during October 1, 2020–March 31, 2022, and April 1, 2022–March 22, 2023, to estimate ratios (slopes) relative to the COVID-19–associated hospital admission rates. To categorize indicators for data visualized on maps, the calculated ratios were used to identify thresholds for each indicator that were anchored to hospital admission rates used in the CCLs (10 and 20 admissions per 100,000 population), but the lower two categories were divided to increase resolution during periods of lower incidence (five, 10, 15, and 20 admissions per 100,000 population). Percent agreement for weekly CCLs and COVID-19 hospital admission levels (<10.0, 10.0–19.9, and ≥20.0 per 100,000 population) was calculated among the 3,220 U.S. counties (and county-equivalent areas) during February 24, 2022–March 23, 2023 (i.e., since the CCLs were launched). The analysis was carried out using Python (version 3.8.6; Python Software Foundation) with packages Pandas (version 1.5.2) and NumPy (version 1.21.6) for all correlations. Linear regression was carried out using scikit-learn (version 1.1.1). This activity was reviewed by CDC and conducted consistent with applicable federal law and CDC policy.^§§^

Normalized trends in surveillance indicators largely aligned over time; differences were observed in lag and proportionality relative to hospital admission rates for both early indicators of COVID-19 activity and disease severity indicators (Figure). Analysis of cross-correlations between surveillance indicators showed that trends in hospital admission rates lagged 1 day behind case rates and 4 days behind the percentages of COVID-19 ED visits and positive test results (either from CELR or NREVSS [i.e., early indicators]) (Table 1). Severity indicators that lagged behind hospital admission rates included inpatient and intensive care unit (ICU) bed occupancy (3–4 days) and deaths (8 days for NVSS and 21 days for aggregate death counts).

**FIGURE:**
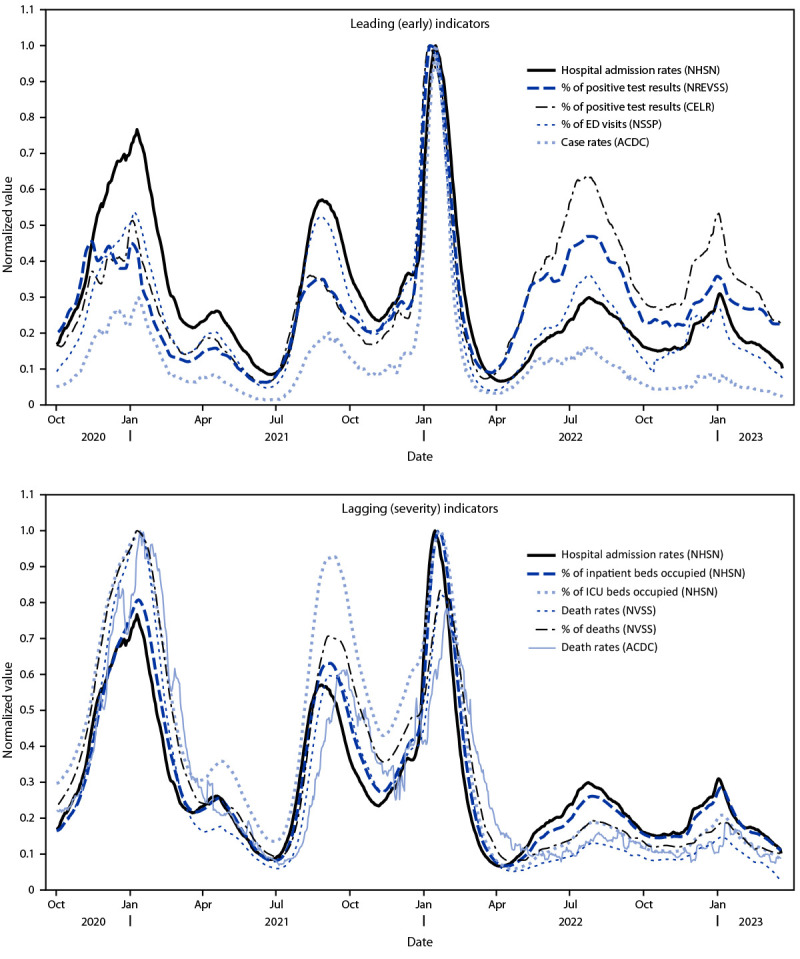
Trends in normalized values* of leading (A) and lagging (B)^†^ COVID-19 surveillance indicators — United States, October 1, 2020–March 22, 2023 **Abbreviations:** ACDC = aggregate cases and death counts; CELR = COVID-19 electronic laboratory reporting; ED = emergency department; ICU = intensive care unit; NHSN = National Healthcare Safety Network; NREVSS = National Respiratory and Enteric Viruses Surveillance System; NVSS = National Vital Statistics System. * Normalized values were obtained by dividing each indicator by its maximum over the displayed time frame, which fixes the peak for each curve at 1. ^†^ Leading or lagging indicators were defined relative to hospital admission rates, which are shown in each panel.

**TABLE 1:** Summary of estimated time lags and ratios for COVID-19 surveillance indicators relative to hospital admission rates per 100,000 population at the state or territory level, by period — United States, October 1, 2020–March 22, 2023

Data (source)	COVID-19 indicator*	Estimated lag (days) vs. hospital admission rates^†^	Ratios with hospital admission rates by period^§^		Category threshold values from May 11, 2023^¶^
		Apr 1, 2022–Mar 22, 2023	Oct 1, 2020–Mar 31, 2022	Apr 1, 2022–Mar 22, 2023	
COVID-19–associated hospitalizations (NHSN)**	Admissions per 100,000 population	0	1.00	1.00	10 and 20
**COVID-19 cases (ACDC)^††^**	Cases per 100,000 population	−1	18.04	15.52	NA
**ED visits for COVID-19 (NSSP)^§§^**	% of all ED visits	−4	0.23	0.31	1.5, 3.0, 4.5, and 6.0
COVID-19–associated hospitalizations (NHSN)**	% of inpatient beds occupied	3	0.43	0.40	2.0, 4.0, 6.0, and 8.0
COVID-19–associated hospitalizations (NHSN)**	% of ICU beds occupied	4	0.86	0.44	2.0, 4.0, 6.0, and 8.0
**COVID-19–associated deaths (NVSS)^¶¶^**	% of all deaths	8	0.66	0.38	2.0, 4.0, 6.0, and 8.0
**COVID-19–associated deaths (NVSS)^¶¶^**	Deaths per 100,000 population	8	0.17	0.07	Auto
**COVID-19–associated deaths (ACDC)^††^**	Deaths per 100,000 population	21	0.13	0.08	NA
Positive SARS-CoV-2 test results (CELR)***	% of positive NAAT rest results	−4	0.49	1.37	NA
**Positive SARS-CoV-2 test results (NREVSS)^†††^**	% of positive NAAT test results	−4	0.48	1.02	5.0, 10.0, 15.0, and 20.0

**Abbreviations:** ACDC = aggregate cases and death counts; Auto = automatic assignment of six categories based on dynamic natural breaks; CCL = COVID-19 Community Level; CELR = COVID-19 electronic laboratory reporting; ED = emergency department; HHS = U.S. Department of Health and Human Services; ICU = intensive care unit; NA = not applicable after May 11, 2023; NAAT = nucleic acid amplification test; NHSN = National Healthcare Safety Network; NREVSS = National Respiratory and Enteric Viruses Surveillance System; NSSP = National Syndromic Surveillance Program; NVSS = National Vital Statistics System.

* Moving 7-day averages were used for all indicators. These averages were available daily for all data sources except NVSS and NREVSS, which were available weekly.

^†^ Cross-correlation was used to calculate the lag (number of days) in indicators relative to hospital admissions as follows: Spearman’s correlations between indicators compared with hospital admission rates per 100,000 population were computed over a moving 12-week window. The offset (ranging from −35 days to 35 days) in indicators that produced the highest mean correlation across all windows and states is displayed. Negative lags correspond to indicators that lead admissions and positive lags correspond to indicators that lag admissions.

^§^ A linear regression model y = mx + b was fit where *x* represents the hospital admission rate, *y* represents the indicator of interest, *m* represents the ratio between indicators, and the intercept *b *was fixed at zero, such that for a one-unit rise in admissions per 100,000 population (*x*), the indicator of interest (*y*) will increase by the ratio value.

^¶^ To categorize indicators for data visualized in maps on COVID Data Tracker, the calculated ratios were used to identify thresholds for each indicator that were anchored to hospital admission rates used in the CCLs (10 and 20 admissions per 100,000 population), but the lower two categories were divided to increase resolution during periods of lower incidence (five, 10, 15, and 20 admissions per 100,000 population).

** As of December 15, 2022, COVID-19 hospital data are required to be reported to CDC’s NHSN, which monitors national and local trends in health care system stress, capacity, and community disease levels for approximately 6,000 hospitals in the United States. Data reported by hospitals to NHSN represent aggregated counts and include metrics capturing information specific to hospital capacity, occupancy, hospitalizations, and admissions. Before December 15, 2022, hospitals reported data directly to HHS or via a state submission for collection in the HHS Unified Hospital Data Surveillance System. Full guidance on hospital reporting and a list of data elements and definitions can be found online. https://www.hhs.gov/sites/default/files/covid-19-faqs-hospitals-hospital-laboratory-acute-care-facility-data-reporting.pdf

^††^ National weekly COVID-19 ACDC data are compiled by CDC using automated data extraction from official jurisdictional data sources (e.g., through application programming interfaces) and direct submissions from jurisdictions. ACDC data shifted from daily to weekly cadence in October 2022, with some jurisdictions continuing to report daily totals and others reporting only weekly totals.

^§§^ NSSP is a collaboration among CDC, local and state health departments, and academic and private partners to collect and analyze electronic health care data, including data from ED visits. NSSP has expanded substantially during the COVID-19 pandemic, with data from 6,300 facilities in all 50 U.S. states, the District of Columbia, and Guam. NSSP includes 75% of all ED visits in the United States. Data are not shown on COVID Data Tracker for states where ED facility participation is low (currently Minnesota and Oklahoma) or diagnosis information is incomplete (currently Missouri). https://www.cdc.gov/nssp/index.html

^¶¶^ NVSS collects and reports mortality statistics using U.S. death certificate data. These data are provided through contracts between CDC’s National Center for Health Statistics and vital registration systems operating in the various jurisdictions legally responsible for the registration of vital events. NVSS data from U.S. territories, other than Puerto Rico, are not included in provisional mortality reporting. https://www.cdc.gov/nchs/nvss/index.htm

*** CELR has become more variable in quality or altogether unavailable in many jurisdictions over time. With the expiration of the COVID-19 public health emergency, HHS can no longer require reporting of negative SARS-CoV-2 laboratory testing results via CELR. https://www.cdc.gov/coronavirus/2019-ncov/lab/reporting-lab-data.html

^†††^ NREVSS collects weekly aggregate SARS-CoV-2 NAAT results from a sentinel network of reporting laboratories in the United States including clinical, public health and commercial laboratories. These data exclude a small proportion of antigen test results and do not include antibody and at-home test results. NREVSS percent positivity data will be added to COVID Data Tracker after May 11, 2023. https://www.cdc.gov/surveillance/nrevss/index.html

Rates of COVID-19–associated hospital admissions and percentages of inpatient beds occupied by COVID-19 patients, deaths that were COVID-19–associated, and ED visits with a diagnosis of COVID-19 had the highest mean correlations for capturing trends across all indicators (Table 2). Hospital admission rates exhibited both a high signal-to-noise ratio and a low geographic consistency, suggesting that this indicator might provide more easily interpretable and reliable information than others. Since October 2020, correlation was strong (*7*) between death rates from NVSS and aggregate death counts (0.79) and between NREVSS and CELR positive test results (0.79); correlation since April 2022 was lower for deaths (0.41) and slightly lower for positive test results (0.70).

**TABLE 2:** Mean correlation with other indicators, autocorrelation (signal-to-noise), and geographic consistency of COVID-19 surveillance indicators — April 1, 2022–March 22, 2023

COVID-19 indicator (data source)	Mean correlation with other indicators (rank)*	Autocorrelation with a 7-day lag (rank)^†^	Geographic consistency metric (rank)^§^
Hospital admissions per 100,000 population (NHSN)^¶^	0.57 (1)	0.92 (3)	0.64 (5)
Percentage of inpatient beds occupied (NHSN)^¶^	0.57 (2)	0.96 (1)	0.73 (10)
Percentage of deaths (NVSS)**	0.56 (3)	0.68 (9)	0.61 (4)
Percentage of ED visits (NSSP)^††^	0.54 (4)	0.90 (5)	0.55 (2)
Percentage of ICU beds occupied (NHSN)^¶^	0.54 (5)	0.89 (6)	0.65 (6)
Deaths per 100,000 population (NVSS)**	0.53 (6)	0.72 (8)	0.67 (7)
Cases per 100,000 population (ACDC)^§§^	0.51 (7)	0.90 (4)	0.58 (3)
SARS-CoV-2 percent positivity (CELR)^¶¶^	0.48 (8)	0.96 (2)	0.73 (9)
SARS-CoV-2 percent positivity (NREVSS)***	0.43 (9)	0.89 (7)	0.70 (8)
Deaths per 100,000 population (ACDC)^§§^	0.29 (10)	0.51 (10)	0.47 (1)

**Abbreviations:** ACDC = aggregate cases and death counts; CCL = COVID-19 Community Level; CELR = COVID-19 electronic laboratory reporting; ED = emergency department; HHS = U.S. Department of Health and Human Services; ICU = intensive care unit; NA = not applicable after May 11, 2023; NAAT = nucleic acid amplification test; NHSN = National Healthcare Safety Network; NREVSS = National Respiratory and Enteric Viruses Surveillance System; NSSP = National Syndromic Surveillance Program; NVSS = National Vital Statistics System.

* Pairwise Spearman’s correlations were used to evaluate associations between indicators after adjusting for lag, and mean correlations were calculated and ranked.

^†^ Autocorrelations (Spearman’s) were used to assess the signal-to-noise ratio for each indicator (compared with itself but offset by 7 days); indicators were ranked based on autocorrelation.

^§^ Geographic consistency was evaluated using a metric calculated by computing daily z-scores for each indicator, averaging these scores by state, and computing the standard deviation over all states. Surveillance indicators with lower values for the geographic consistency metric are less likely to have jurisdictions consistently reporting indicator values higher or lower than the average. Indicators were ranked based on their geographic consistency metric.

^¶^ As of December 15, 2022, COVID-19 hospital data are required to be reported to CDC’s NHSN, which monitors national and local trends in health care system stress, capacity, and community disease levels for approximately 6,000 hospitals in the United States. Data reported by hospitals to NHSN represent aggregated counts and include metrics capturing information specific to hospital capacity, occupancy, hospitalizations, and admissions. Before December 15, 2022, hospitals reported data directly to HHS or via a state submission for collection in the HHS Unified Hospital Data Surveillance System. Full guidance on hospital reporting and a list of data elements and definitions can be found online. https://www.hhs.gov/sites/default/files/covid-19-faqs-hospitals-hospital-laboratory-acute-care-facility-data-reporting.pdf

** NVSS collects and reports mortality statistics using U.S. death certificate data. These data are provided through contracts between CDC’s National Center for Health Statistics and vital registration systems operating in the various jurisdictions legally responsible for the registration of vital events. NVSS data from U.S. territories, other than Puerto Rico, are not included in provisional mortality reporting. https://www.cdc.gov/nchs/nvss/index.htm

^††^ NSSP is a collaboration among CDC, local and state health departments, and academic and private partners to collect and analyze electronic health care data, including data from emergency department visits. NSSP has expanded substantially during the COVID-19 pandemic, with data from 6,300 facilities in all 50 U.S. states, the District of Columbia, and Guam. NSSP includes 75% of all emergency department visits in the United States. Data are not shown on COVID Data Tracker for states where ED facility participation is low (currently Minnesota and Oklahoma) or diagnosis information is incomplete (currently Missouri). https://www.cdc.gov/nssp/index.html

^§§^ National weekly COVID-19 ACDC data are compiled by CDC using automated data extraction from official jurisdictional data sources (e.g., through application programming interfaces) and direct submissions from jurisdictions. ACDC data shifted from daily to weekly cadence in October 2022, with some jurisdictions continuing to report daily totals and others reporting only weekly totals.

^¶¶^ Percent positivity from CELR have become more variable in quality or altogether unavailable in many jurisdictions over time. With the expiration of the COVID-19 public health emergency, HHS can no longer require reporting of negative SARS-CoV-2 laboratory testing results via CELR. https://www.cdc.gov/coronavirus/2019-ncov/lab/reporting-lab-data.html

*** NREVSS collects weekly aggregate SARS-CoV-2 NAAT results from a sentinel network of reporting laboratories in the United States including clinical, public health and commercial laboratories. These data exclude a small proportion of antigen test results and do not include antibody and at-home test results. NREVSS percent positivity data will be added to COVID Data Tracker after May 11, 2023. https://www.cdc.gov/surveillance/nrevss/index.html

Estimated ratios for percentage of positive test results relative to hospital admission rates from both CELR and NREVSS have increased since April 2022 related to decreased testing volumes, although CELR was more affected, possibly related to differential reporting of negative results (Table 1) (Figure).^¶¶^ The ratios for percentage of ICU beds occupied by COVID-19 patients, percentage of deaths that are COVID-19–associated, and rates of COVID-19 deaths have decreased relative to hospital admissions since April 2022, likely due to decreased severity of recent infections as a consequence of high population levels of vaccine- and infection-induced immunity, improvements in medical treatment, and changes in variants over time.

A comparison of CCL and COVID-19 hospital admission level designations (low, medium, or high) by week during February 2022–March 2023 demonstrated >99% concordance among 3,220 counties (Supplementary Figure, https://stacks.cdc.gov/view/cdc/127731). Most discordant levels were reported during periods of high COVID-19 incidence during February and March 2022. When the levels were discordant, CCLs exceeded the hospital admission levels.

## Discussion

This evaluation of national COVID-19 surveillance data sources and indicators was performed in anticipation of the transition from the COVID-19 pandemic response to routine public health activities that require sustainable sources of surveillance data and reliable indicators after the end of the public health emergency declaration on May 11, 2023. The evaluation determined that hospital admission rates are a suitable and timely primary indicator for monitoring COVID-19 trends. Using COVID-19 mortality data from NVSS improves timeliness for monitoring disease severity by up to 13 days. Leading indicators such as the percentage of ED visits with a COVID-19 diagnosis and percentage of positive SARS-CoV-2 test results can capture changes in trends approximately 4 days earlier than hospital admission rates and provide complementary monitoring information, albeit with more limited geographic coverage.

COVID-19–associated hospital admission rates are available down to the level of the health service area, which is mapped to counties (*1*). The high concordance with CCLs is not surprising, because COVID-19 hospital admissions are the primary driver of CCLs and apply identical threshold levels, ensuring continuity beyond the public health emergency. One limitation of the existing level thresholds is insufficient granularity to detect changes during periods of low incidence; further monitoring and analysis would be needed before adjusting thresholds.

Early in the pandemic, aggregate death reporting provided more up-to-date death counts than did NVSS, but timeliness for the two systems has become more similar over time because of improvements in NVSS death certificate data processing (*8*). Analysis of the NVSS data by date of death makes the impact of reporting delays on recent deaths more apparent than aggregate death data by date of report (i.e., backfill death counts are assigned to recent report dates rather than the dates when the deaths occurred). However, NVSS data elements are more complete (e.g., for race and ethnicity), and the percentage of COVID-19 deaths from NVSS is not biased by incomplete reporting in recent weeks because death certificate data from COVID-19 and all causes have similar timeliness (*4*).

Over the course of the pandemic, the NSSP network has expanded considerably with ED visit data available for most jurisdictions (*1*). The data source for percentage of positive SARS-CoV-2 test results will change from CELR to NREVSS after the public health emergency declaration expires and will be reported at the regional level because of limited numbers of reporting laboratories in some states (*1*). Voluntary reporting to NREVSS has been used for many years to track the percentage of positive test results for numerous respiratory viruses including influenza and respiratory syncytial virus (*5*).

The findings in this report are subject to at least three limitations. First, it was not possible to distinguish between lags related to time to event (e.g., time from infection until death) and reporting delays. Further, retrospective findings do not account for reporting lags affecting recent data or potential future changes to reporting cadence (e.g., change from daily to weekly reporting), including for hospitalization data (*1*). As such, the lags presented serve as lower bounds on the effective lag when using these data for real-time monitoring, especially for recent weeks with incomplete reporting. Second, data availability is changing with the end of public health emergency declaration on May 11, 2023, and data availability and quality will likely continue to change over time, potentially affecting their utility for COVID-19 monitoring. The current analysis focused on available data sources moving forward. Finally, this national evaluation used states and territories as a geographic unit of analysis, but findings might vary by jurisdiction based on geographic heterogeneity. This report can serve as a model for similar evaluations that could be undertaken at state levels.

COVID-19 hospital admission rates from NHSN are a timely and suitable primary indicator for monitoring trends in COVID-19 activity. Using the percentage of COVID-19 deaths from NVSS will allow more timely monitoring of COVID-19 severity and mortality trends. The percentage of COVID-19 ED visits and percentage of positive test results can serve as early indicators for COVID-19 trend monitoring. Collectively, these surveillance data sources and indicators can support monitoring of the impact of COVID-19 and related prevention and control strategies as ongoing public health priorities.

**Summary**

**What is already known about this topic?**

COVID-19 monitoring will remain a public health priority after the U.S. public health emergency declaration expires on May 11, 2023.

**What is added by this report?**

Assessment of available surveillance indicators found that COVID-19 hospital admission levels were concordant with COVID-19 Community Levels. COVID-19–associated hospital admission rates lagged 1 day behind case rates and 4 days behind percentages of COVID-19 emergency department visits and positive SARS-CoV-2 test results. National Vital Statistics System trends in the percentage of COVID-19 deaths strongly correlated with, and were 13 days timelier, than aggregate death count data.

**What are the implications for public health practice?**

Rates of COVID-19–associated hospital admission and the percentages of positive test results, COVID-19 emergency department visits, and COVID-19 deaths are suitable and timely indicators of trends in COVID-19 activity and severity.

## Footnotes

* These authors contributed equally to this report.

† As of December 15, 2022, COVID-19 hospital data are required to be reported to CDC’s NHSN, which monitors national and local trends in health care system stress, capacity, and community disease levels for approximately 6,000 hospitals in the United States. Data reported by hospitals to NHSN represent aggregated counts and include metrics capturing information specific to hospital capacity, occupancy, hospitalizations, and admissions. Before December 15, 2022, hospitals reported data directly to the U.S. Department of Health and Human Services (HHS) or via a state submission for collection in the HHS Unified Hospital Data Surveillance System.

§ CCLs are a composite metric using COVID-19 hospital admissions per 100,000 population, the percentage of inpatient beds occupied by COVID-19 patients from NHSN, and COVID-19 cases per 100,000 population from aggregate case reporting at the county or county-equivalent level. Because of the choice of thresholds for each of these data elements, the hospital admissions rate is the primary determinant of the CCL for any county, and CCLs for each county will be as high as or higher than the corresponding COVID-19 hospital admission level (i.e., categorized using identical rate thresholds).

¶ NVSS data also contained suppressed values for areas and weeks with counts between 1 and 9. Suppressed counts were treated as 1 when calculating percentage of deaths and deaths per 100,000 population. A sensitivity analysis that replaced suppressed counts with 9 obtained similar results.

** Negative lags correspond to indicators that precede hospital admissions, and positive lags correspond to indicators that follow admissions. Null values were omitted during these calculations.

†† This model took the form y = mx + b where *x* represents the hospital admission rate, *y* represents the indicator of interest, the slope *m* represents the ratio between indicators, and the intercept *b* was fixed at zero. In this formulation, the ratio can be interpreted as the increase in the indicator of interest (*y*) corresponding to a one-unit increase in admissions per 100,000 population (*x*).

§§ 45 C.F.R. part 46.102(l)(2), 21 C.F.R. part 56; 42 U.S.C. Sect. 241(d); 5 U.S.C.0 Sect. 552a; 44 U.S.C. Sect. 3501 et seq.

¶¶ The CELR data have become more variable in quality or altogether unavailable in many jurisdictions over time.
